# UBER: UAV-Based Energy-Efficient Reconfigurable Routing Scheme for Smart Wireless Livestock Sensor Network

**DOI:** 10.3390/s22166158

**Published:** 2022-08-17

**Authors:** Mohammed A. Alanezi, Abdulazeez F. Salami, Yusuf A. Sha’aban, Houssem R. E. H. Bouchekara, Mohammad S. Shahriar, Mohammed Khodja, Mostafa K. Smail

**Affiliations:** 1 Department of Computer Science and Engineering Technology, University of Hafr Al Batin, Hafr Al Batin 31991, Saudi Arabia; 2 Department of Computer Engineering, University of Ilorin, Ilorin 240103, Nigeria; 3 Department of Electrical Engineering, University of Hafr Al Batin, Hafr Al Batin 31991, Saudi Arabia; 4 Department of Electronics, College of Engineering, Mustaqbal University, Buraidah 51452, Saudi Arabia; 5 Department of Electrical Engineering, Faculty of Technology, M’sila University, M’sila 28000, Algeria; 6 Institut Polytechnique des Sciences Avancées, 63 Boulevard de Brandebourg, 94200 Ivry-sur-Seine, France

**Keywords:** cattle, herd cluster-based routing, performance analysis, unmanned aerial vehicle, wireless livestock sensor network

## Abstract

This paper addresses coverage loss and rapid energy depletion issues for wireless livestock sensor networks by proposing a UAV-based energy-efficient reconfigurable routing (UBER) scheme for smart wireless livestock sensor networking applications. This routing scheme relies on a dynamic residual energy thresholding strategy, robust cluster-to-UAV link formation, and UAV-assisted network coverage and recovery mechanism. The performance of UBER was evaluated using low, normal and high UAV altitude scenarios. Performance metrics employed for this analysis are network stability (NST), load balancing ratio (LBR), and topology fluctuation effect ratio (TFER). Obtained results demonstrated that operating with a UAV altitude of 230 m yields gains of 31.58%, 61.67%, and 75.57% for NST, LBR, and TFER, respectively. A comparative performance evaluation of UBER was carried out with respect to hybrid heterogeneous routing (HYBRID) and mobile sink using directional virtual coordinate routing (MS-DVCR). The performance indicators employed for this comparative analysis are energy consumption (ENC), network coverage (COV), received packets (RPK), SN failures detected (SNFD), route failures detected (RFD), routing overhead (ROH), and end-to-end delay (ETE). With regard to the best-obtained results, UBER recorded performance gains of 46.48%, 47.33%, 15.68%, 19.78%, 46.44%, 29.38%, and 58.56% over HYBRID and MS-DVCR in terms of ENC, COV, RPK, SNFD, RFD, ROH, and ETE, respectively. The results obtained demonstrated that the UBER scheme is highly efficient with competitive performance against the benchmarked CBR schemes.

## 1. Introduction

Livestock farming (LF) is one of the global economy’s backbone industries, but the growing world population has placed tremendous pressure on the demand for food [1,2,3]. This implies that livestock industries’ production capacity and efficiency must scale up to meet this increasing food demand [3,4,5]. Industrialists have explored arrays of wireless sensor network (WSN) technologies to improve the quality, quantity and efficiency of LF [1,6,7,8,9]. Recently, the performance of Internet of things (IoT) devices equipped with embedded sensor nodes (SNs) have been significantly enhanced, which has led to effective control and management of distributed energy supply systems (DESS) [3,10,11]. The DESS acts as an energy source for the WSN while monitoring the livestock’s movement, activities, and health status [2,3]. It must be mentioned that wearable SNs used for smart livestock monitoring applications are portable battery-constrained devices with information gathering and processing capabilities [3,10,12]. However, with harsh weather conditions, the dynamic and random movement of livestock in the field makes livestock monitoring applications based solely on wearable/strap-on SNs face challenges. These challenges include the high cost of frequent SN replacement, erratic loss of coverage, short network lifetime, rapid energy drainage, and the frequent need for human involvement in the system’s operation.

These challenges have prompted researchers to explore unmanned aerial vehicle (UAV)-aided solutions to address routing issues associated with monitoring livestock movement and activities [11,13,14,15,16,17,18,19]. UAV-aided solutions have created the possibility of designing cost-effective livestock surveillance solutions [2,20,21,22,23,24]. In such surveillance solutions, UAVs serve as: 1.) sinks for collecting time-sensitive data from strap-on SNs and 2.) mobile aerial stations for directly monitoring the livestock [25,26]. Therefore, the massive production of LF products requires innovative integration of WSN and UAV technologies to address the increasing global food demand challenge. Several integrated UAV-WSN solutions have been deployed to increase the yield of LF products, where UAVs act as mobile sinks (MS) collecting livestock data from wearable SNs, and various artificial intelligence (AI) tools are employed for interactive data analytics [3,8,25]. These integrated solutions help increase sales revenue, sustain large-scale production, and ensure the distribution of quality LF products to the end consumers [3,11,26]. Most importantly, extensive unsupervised livestock monitoring applications address the difficulty, monotony, and time-consuming challenges of manual monitoring of large livestock farms. However, challenges of network lifetime issues and frequent disruptive topology changes still exist and demand further research.

Researchers have proffered various generic [27,28,29,30,31,32,33,34] and specific [3,25,35,36,37,38,39,40,41,42,43] routing algorithms to address these challenges. Among the proposed approaches, cluster-based routing (CBR) techniques have shown more computational simplicity, versatility, robustness and effectiveness in reducing energy consumption and preserving network connectivity/coverage [3,44,45,46].

Effective management of unattended and large LF fields requires energy-efficient routing techniques and scalable network architectures. This is usually achieved by logically organizing wearable SNs attached to livestock herds into groups called herd clusters (HCs) in this context to achieve energy efficiency and network scalability. From each HC, some set of SNs are elected as HC leads (HCLs) based on their residual energy, relative access and proximity to the MS. HC members (HCMs) transmit livestock data to their HCLs by using single-hop or multi-hop transmission mode. Subsequently, HCLs forward aggregated data to the aerial MS for onward transmission to the base station (BS) [41,43,47,48].

Based on this herd cluster-based routing strategy, this paper presents a UAV-based energy-efficient reconfigurable (UBER) routing algorithm for smart wireless livestock sensor networking applications. UBER relies on a dynamic residual energy thresholding mathematical model, robust cluster-to-UAV link formation strategy, and UAV-assisted network coverage and recovery mechanisms. Simulation experiments were carried out with OMNET++ and MATLAB, while comparative performance analysis was performed with respect to hybrid heterogeneous routing (HYBRID) and mobile sink using directional virtual coordinate routing (MS-DVCR) techniques. The results obtained demonstrated that the proposed UBER scheme is highly energy-efficient with competitive network performance when benchmarked against existing CBR schemes.

The structure of the remainder of this paper is organized in the following manner: Section 2 covers the related research works pertinent to CBR approaches for wireless livestock sensor networking applications. Section 3 provides a technical description of the proposed UBER protocol, while Section 4 presents the obtained simulation results and supporting discussions. Section 5 concludes this paper.

## 2. Related Works

Traditional CBR techniques are classified as generic based on their mode of network operation and applicability to a wide range of WSN applications. However, generic CBR techniques often suffer from single HCL failure issues, high energy consumption due to single-hop long-range data transmission, high time complexities, and cluster size challenges [27,28,29,30,31,32,33,34,44,45,49,50,51].

The grey wolf optimizer (GWO)-based technique was proffered as a nature inspired CBR algorithm for ensuring seamless and robust data transmission in livestock monitoring applications [3]. This algorithm relies on the behavioral pattern of gray wolves and key network parameters, such as transmission range, cluster size, residual energy and data route to minimize energy consumption [3]. The limitation of this algorithm is that the HC configuration process is not flexible and adaptable to varying network requirements.

The Markov decision process (MDP)-based technique was proposed as a path planning CBR technique that utilizes single UAV and static SNs for HC monitoring and data gathering in a large LF field [25]. The CBR technique relies on Markov decision process together with reward and value of information maximization analytical models. The key benefit of this technique is the significant reduction in message delay [25]. The major drawbacks of this technique are the algorithmic computational cost and high energy tax.

The dynamic decentralized/centralized free conflict unmanned aerial vehicle (DDCFC-UAV) technique was proposed as a security-oriented CBR scheme that relies on SNs mounted on UAVs to monitor a defined LF field [35]. The predefined LF field is logically categorized into virtual HCs, and UAVs are assigned to monitor their assigned HC zones. HCLs are elected by the UAV at each HC zone using dynamic network and energy requirements criteria stipulated by the CBR scheme [35]. The drawback of this CBR scheme is the challenge of maintaining connectivity for the multi-UAV communication architecture.

The hybrid heterogeneous routing (HYBRID) technique was proffered as a network lifetime improvement CBR technique by employing SNs deployed in harsh LF environments where energy efficiency is achieved by dividing the LF field into HCs and placing the location of the BS at the edge of the LF field [36]. The criteria for HC formation are the residual energy of SNs, which gives SNs having higher residual energy more chance of being elected as HCLs. Inter-HC distance is also considered as a factor for multi-hop data transmission of livestock data in the network [36]. The drawback of this CBR technique is the algorithmic complexity associated with switching between varying energy levels and different modes of transmission.

The mobile sink using directional virtual coordinate routing (MS-DVCR) technique was offered as an energy minimization geographic routing scheme that relies on a directional virtual coordinate strategy in conjunction with the aerial MS operation [37]. The central objective of this scheme is to reduce the frequency of network updates transmitted by the MS to a BS. The main advantage of this scheme is that it provides an alternative solution for dealing with MS localization without carrying out physical distance measurements [37]. The major limitation of this scheme is the high overhead tied to maintaining and exchanging information related to the virtual coordinates within the network.

The lightweight dynamic clustering algorithm (LDCA) was proposed as a real-time low-complexity dynamic CBR scheme for livestock monitoring applications having limited processing resources [38]. This CBR scheme utilizes single-hop transmission, variable cluster size, and multiple parameters (SN-to-MS distance, signal strength, residual energy, noise level, environmental factors) for HC configuration to maximize network coverage [38]. The drawbacks of this scheme are SN buffering and regional separation issues associated with rapid MS mobility patterns.

## 3. Proposed UBER Scheme

This section discusses the integrated UAV-WSN heterogeneous network model, fundamental assumptions, cluster configuration, and data gathering phases.

### 3.1. Integrated UAV-WSN Network Model

**Figure 1:**
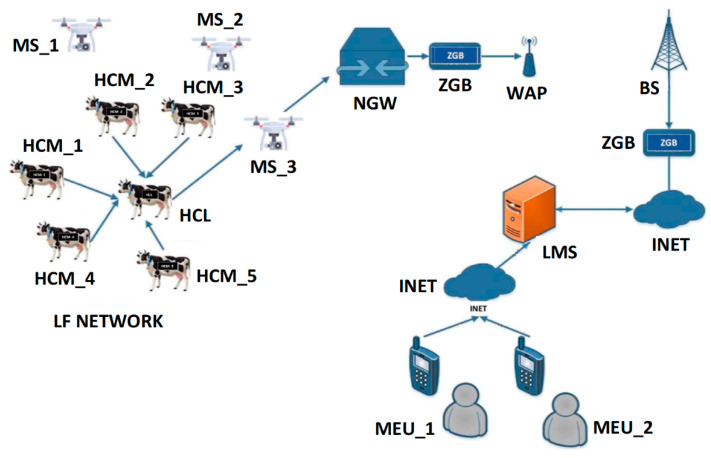
UBER Network Model.

Figure 1 shows the proposed UAV-WSN integrated solution for livestock monitoring applications. In this network model, strap-on SNs acting as HCMs monitor vital parameters (location, perspiration, temperature, heart rate) and transmit sensed parameters to their respective HCLs. The HCLs aggregate all sensed parameters received from their respective HCMs and relay the compressed data to the neighboring UAVs acting as MS. UAVs fly over the LF network perimeter at an altitude within the coverage of the SNs. These MS entities forward aggregated data to the livestock monitoring server (LMS) for processing through a network gateway (NGW)-BS link enabled with ZigBee (ZGB) interface. The LMS analyzes the received livestock data and triggers the required LF controller (LFC) devices, such as temperature regulators, alarms, lighting controllers, and other LFC devices. Authorized mobile end users (MEUs) can check and work on livestock data from the LMS via Internet (INET) connection.

### 3.2. Assumptions

The fundamental assumptions for the proposed UBER CBR scheme are:

- All SNs are wearable, portable, and identical, with similar energy resources, processing capacities and transceiver characteristics. This assumption describes the physical and electronic properties of the sensor nodes suitable for livestock (cattle) monitoring. It is necessary for the sensors to be wearable and portable to prevent discomfort to the livestock, facilitate sensing through direct body contact, and make sensor replacement easier. It is necessary for the sensors to be identical to avoid tagging/stamping, synchronization and mismatch errors during data aggregation and processing.
- UAVs act as MS with more onboard radios and higher energy resources, processing capacities and transceiver range. This assumption describes the functional and electronic properties of the UAVs suitable for the integrated model presented in Figure 1. The fewer UAVs deployed should have more onboard radios to accommodate the influx of periodic traffic from different clusters.
- UAV’s spatial movement is 3-D with a variable velocity profile. This assumption describes the spatial motion capability (in x, y, z direction following straight-line left-to-right up-and-down scanning pattern) of the employed UAV and its limitations (no axial rotation, no angular twists/bends). Velocity profiles for the UAV are stationary, scanning velocity (20 m/s), and data gathering velocity (5 m/s) as stated in Table 1.
- UAVs have embedded intelligence for smart decision-making, and they can be controlled from the LMS. This assumption describes the cognitive capability of the UAV and its limitations (not fully autonomous as its activities can be controlled from the LMS). The reason for utilizing this semi-autonomous arrangement is to prevent out-of-perimeter straying, which can lead to lost UAV, theft, unrestrained energy loss or device damage.

### 3.3. Energy Consumption Model

The traditional energy consumption model is centrally dependent on the transmission distance (*l*) and the number of packet bits transferred (*b*), as postulated in [27,28] as the first-order radio energy model. The implication of this is that the transceiver energy tax (*E*_TAX_) increases exponentially with increasing transmission distance, as expressed in Equation (1):

(1) $$E_{TAX}=b\cdot \left[E_{EC}+\left(E_{PA}\cdot l^{2}\right)\right]$$

where, *E*_EC_ and *E*_PA_ are the circuity energy dissipation and amplifier-dependent energy loss parameter, respectively. Equation (2) defines the transmission distance as [52]:

(2) $$l={\left[\frac{\lambda }{16\pi^{2}}\right]}^{\frac{1}{\alpha }}$$

where, *α* is the path loss exponent and *λ* is the wavelength. It must be mentioned that the free space path loss model has limitations for SN-to-UAV communication, especially in the presence of obstacles and weather conditions, which can affect the path loss and increase the path loss exponent. This is one of the technical reasons why the UAV’s normal altitude is operated at 230 m, which falls within the SN’s transmission range of 250 m in order to preserve network coverage and connectivity. Furthermore, multipath communication has been incorporated into an upcoming sequel paper in order to serve as an improvement. UBER’s proposed energy consumption model curbs this exponential increase in *E*_TAX_ by forming HCs based on the HCM-to-HCL and HCL-to-MS distance parameter estimates, which allows SNs nearest to the MS to be elected as HCLs. The effectiveness of this energy conservation strategy is enhanced with the use of dynamic residual energy thresholding ($E_{TAX}^{th}$) technique as:

(3) $$E_{TAX}^{th}=\left\{\begin{array}{c} b\cdot \left[E_{EC}+\left(E_{S}\cdot l\right)\right],\;if\;l\leq l_{th}\\ b\cdot \left[E_{EC}+\left(E_{L}\cdot l\right)\right],\;if\;l>l_{th} \end{array}\right.$$

where, *E*_S_, *E*_L_, and *l*_th_ are short-range energy transmission, long-range energy transmission and distance threshold. *E*_S_ and *E*_L_ are approximated based on practical design considerations for IEEE 802.15.4 RF transceivers [53,54]. The distance threshold is defined as:

(4) $$l_{th}=\frac{2\cdot f_{c}\cdot h_{tx}\cdot h_{rx}}{k\cdot v}$$

where, *k*, *f*_c_, *v*, *h*_tx_, and *h*_rx_ are threshold constant, carrier frequency, signal velocity, transmitting SN antenna height and receiving SN antenna height, respectively. It must be mentioned that Equation (3) has been simplified to a linear form (with the use of practical field approximations [54]) compared to the quadratic form in Equation (1). This adaptive/programmable output power level specification helps to prevent exponential *E*_TAX_ increase, ensures robust HCL-to-MS data transmission, and reduces distance-dependent interference experienced by SNs in the LF network perimeter.

### 3.4. Cluster Configuration Phase

The proposed UBER CBR algorithm commences with the network discovery phase. The clustering costs (*CC*_x_) based on *Z*_max_ (peak value of the received signal strength levels) are shared among neighboring SNs through HELLO packets. Z_max_ is the peak of the received signal power (RSSI) values, which is a reliable indicator of the signal strength. RSSI interpretation is based on proximity as relatively higher RSSI values will be recorded for closer nodes. *CC*_x_ is in dBm and it is formulated as:

(5) $$CC_{x}=max\left[Z_{x,t}\right]$$

where, *x* and *Z*_x,t_ denote each SN and received signal strength level for SN_x_ obtained from UAV signals at duration *t,* respectively. After the network discovery phase, distributed repetitive procedures are used to elect HCLs from candidate SNs. SNs with an established connection with the MS opt to become HCLs by setting their electability probability (*HCL*_PR_) as:

(6) $$HCL_{PR}=\left\{\begin{array}{c} max\left[LIM_{UP}\cdot \frac{E_{rsd}}{E_{tot}},LIM_{LOW}\right],\;if\;CC_{x}>0\\ 0,\;\;\;\;\;\;\;\;\;\;\;\;\;\;\;\;\;\;\;\;\;\;\;\;\;\;\;\;\;\;\;\;\;\;\;\;\;\;\;\;\;\;\;\;\;\;\;\;\;\;\;\;\;\;if\;CC_{x}=0 \end{array}\right.$$

where, *LIM*_UP_, *LIM*_LOW_, *E*_rsd_, and *E*_tot_ are upper limit probabilistic values for HCL contentions, lower limit probabilistic values for HCL contentions, residual energy, and total energy, respectively. LIM_UP_ is the upper limit set in the network to limit number of HCL competitions/announcements while LIM_LOW_ is the lower limit. Selection of these two parameters is adjusted to allow rapid convergence of HCL_PR._ *E*_rsd_ is obtained formally as:

(7) $$E_{rsd}=E_{tot}-E_{TAX}^{th}$$

*E*_rsd_/*E*_tot_ ratio incorporates dynamic residual energy thresholding into the HC configuration process. The formulated electability probability implies that SNs with higher *E*_rsd_ and established proximal connection to the MS will have a higher chance of being elected as trial HCLs after each round (*R*), 1 < *R* < *R*_max_. *R* means a round of network operation. INFO packets about the trial HCLs are exchanged with neighboring SNs to maintain a set of neighboring trial HCLs. An ordinary node SN_x_ selects its HCL (MY_HCL) based on the trial HCL with the least *CC*_x_ in its neighboring set. The newly elected HCL broadcast POLLING packets, HCL_polling(SN_ID, trial_HCL, *CC*_x_) to its neighboring SNs. After successful completion of the network operation round, the status of the trial HCL is configured to final HCL and POLLING packets, HCL_polling(SN_ID, final_HCL, *CC*_x_), are broadcasted to neighboring SNs. SNs receiving the POLLING packets respond with JOIN packets to update their cluster membership information. At the end of the set-up phase, SNs are either tagged with HCM or final HCL status, while orphaned SNs establish a connection with the closest HCM to become an affiliate of the HC. This cluster arrangement ensures that SN-to-MS data transmission requires a maximum of three hops in the worst-case scenario of orphaned SNs. The cluster configuration algorithm is shown in Algorithm 1.
**Algortihm 1** Cluster Configuration Algorithm for UBER.1:  **for** each SN_x_ received MS_CONNECT signal2:    **if** (Z_x,t_ ← MS && Z_x,t_ ≠ NULL)3:      status.connect(MS) ← TRUE4:      compute CC_x_ ← find_peak(Z_x,t_)5:    **else**6:      status.connect(MS) ← FALSE7:      CC_x_ ← −INF8:    **end if**9:    broadcast CCx to SNx.ADJ within CTR10:    status.final_HCL ← FALSE11:  **end for**12:    **while** (R ≠ R_max_ && HCL_PR_ ≠ 1)13:      **if** (status.connect(MS) ← TRUE && status.trial_HCL ← TRUE)14:        HHCL_PR_ ← rand(0,1)CL_PR_ ← rand(0,1)15:        elect.MY_HCL ← trial_HCL.min(CC_x_)16:        **if** (MY_HCL = SN_ID && HCL_PR_ ≠ 1)17:          broadcast HCL_polling(SN_ID, trial_HCL, CC_x_)18:          status.final_HCL ← FALSE19:
        **end if**
20:      **else**
21:        broadcast HCL_polling(SN_ID, final_HCL, CC_x_)22:
      **end if**
23:      HCL_PR_ at t−1 ← HCL_PR_24:      HCL_PR_ ← min(2xHCL_PR_,1)25:    **end while**26:   status.final_HCL ← TRUE27:   update(trial_HCL) ← POLLING packet28:   elect(trial_HCL) ← IDLE29:   elect(final_HCL) ← ACTIVE30:   broadcast POLLING packet within CTR31:      **for** each ordinary SN_x_ received POLLING packet32:          compute Euclidean distance cost33:          multicast JOIN packet34:
      **end for**
35:      **if** SN_x_ did not receive POLLING packet36:      compute euclidean distance cost to SN_x_.ADJ within CTR37:      construct EDGE using least distance cost38:
      **end if**
39:      **for** each HCL40:          register HCM list41:          construct EDGE with HCM set42:
      **end for**


### 3.5. Data Gathering Phase

In the steady-state phase, the radio of each HCM is triggered to WAKE state for monitoring and transmitting livestock data to their respective HCLs. It must be mentioned that SNs are in WAKE state only for active network operation time and are put into SLEEP state otherwise. The HCLs are assigned the network task of data aggregation and forwarding to the nearest MS. Multi-hop communication chain is relied upon for end-to-end (HCM-to-HCL, HCL-to-MS, and MS-to-LMS) data transmission. After the LMS receives all the desired livestock data, END_ROUND packet is broadcasted to the network by the HCLs based on the interrupting signal received from the MS.

## 4. Results and Discussions

This section discusses UBER’s performance metrics, simulation parameters, algorithmic complexity, performance analysis to different MS altitudes, and comparative performance evaluation of UBER against HYBRID and MS-DVCR.

### 4.1. Performance Metrics

The measures employed for performance analysis are network coverage (COV), network stability (NST), energy consumption (ENC), received packets (RPK), topology fluctuation effect ratio (TFER), SN failures detected (SNFD), route failures detected (RFD), routing overhead (ROH), load balancing ratio (LBR), and end-to-end delay (ETE).

### 4.2. Simulation Parameters

Simulation experiments for this research work were conducted with OMNET++ and MATLAB. Selected key parameters employed for this simulation are provided in Table 1. From Table 1, energy tax threshold levels are obtained from Equation (3) and their significance is explained as step-wise programmable power levels adopted to prevent exponential *E*_TAX_ increase. Aggregation energy is the energy consumed to perform data aggregation by the HCL in order to reduce redundancy before onward transmission to the MS. Dual frequency (433 MHz for SN localization and handshaking and 2.4 GHz for data transmission) are used. The PHY/MAC layer characteristics are based on IEEE 802.15.4 protocol specifications while the NETWORK layer characteristics is based on ZigBee protocol specifications (as indicated in Figure 1). Command and control signaling is used to enable the sending of a command signal to the UAV from the LMS and receiving data traffic from the UAV payload. Due to the focus of this paper on a smart wireless livestock sensor network, the SNs deployment is by attachment to the neck region of the livestock (as shown in Figure 1) and the UAVs are deployed to follow the livestock herd within the LF network perimeter.

### 4.3. Algorithmic Complexity

Through simulation experiments, it was observed that when *LIM*_LOW_ is adjusted to 0.005, UBER converges at around 11 iterative rounds. Furthermore, it was also observed that if *LIM*_UP_ is adjusted to 0.05 (i.e., HCL of 5%), UBER converges at around seven iterative rounds, together with the simultaneous observation that *HCL*_PR_ converges to one after seven iterative rounds. Therefore, this shows that UBER successfully converges after a constant value of iterative rounds, and consequently, it has algorithmic complexity of *Θ* (1).

### 4.4. Performance Analysis

#### 4.4.1. Analysis of UBER Performance

To examine the variations in MS altitude with respect to network stability, load balancing/distribution and topology fluctuation effect on connectivity on UBER performance, scenarios of low altitude (120 m), normal altitude (230 m), and high altitude (340 m) were experimented within the simulation. This choice of normal altitude (230 m) is duly guided by practical network design considerations. This normal altitude falls within the SN’s transmission range of 250 m and lies above the close range of the SNs.

#### 4.4.2. Effect of MS Altitude on Network Stability (NST)

**Figure 2:**
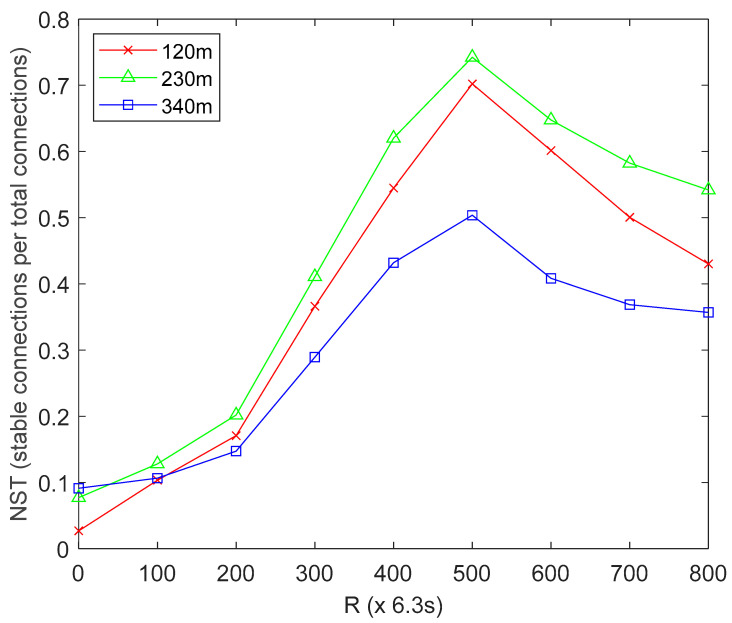
Effect of MS Altitude on Network Stability.

NST is defined as the ratio of the number of stable HCL-to-MS connections to the total number of connections after *R* network operation rounds. From Figure 2, the red line represents UBER’s network stability performance trend under low MS altitude of 120 m, the green line represents UBER’s network stability performance trend under normal MS altitude of 230 m, and the blue line represents UBER’s network stability performance trend under high MS altitude of 340 m. With reference to Figure 2, a higher NST value (NST ≥ 0.5) is desired as this means the MS can maintain a connection with the HCL for a desirably long period (after *R* network operation rounds) before losing the connection. The network recorded an average NST of 0.3004, 0.5391 and 0.3829 for the high-altitude (indicated as blue line), normal-altitude (indicated as green line) and low-altitude (indicated as red line) scenarios throughout network operation. This means that by operating the MS at normal altitude (indicated as green line), the network gains NST of 31.58% and 12.79% over a similar network configuration operating at high altitude (indicated as blue line) and low altitude (indicated as red line), respectively. The technical justification for this is that operating at high altitude (indicated with blue line) keeps the MS almost out of communication range; while operating at low altitude (indicated with red line) is not feasible due to the LF network terrain, interference, collision and congestion issues. Results in Figure 2 emphasize the importance of adopting a suitable MS altitude on NST performance.

#### 4.4.3. Effect of MS Altitude on Load Balancing Ratio (LBR)

**Figure 3:**
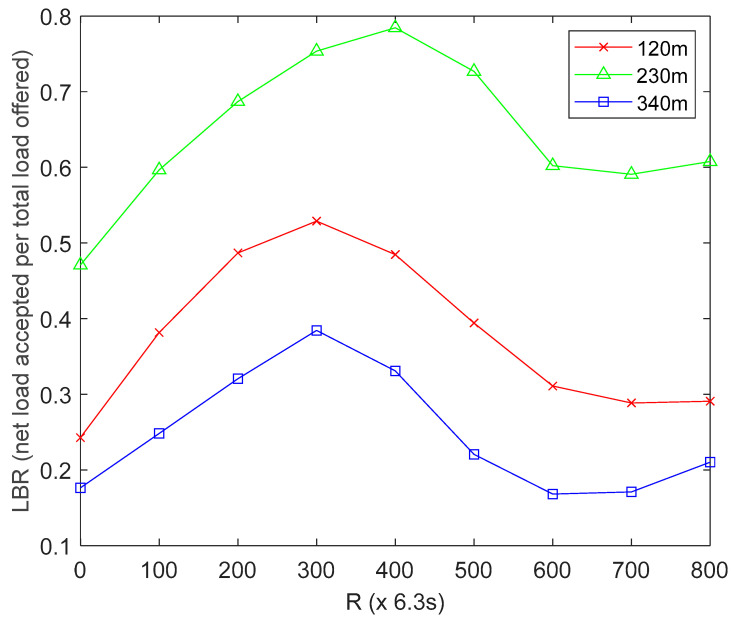
Effect of MS Altitude on Load Balancing Ratio.

The LBR is defined as the comparative ratio of net load successfully accepted by the MS to the total load offered by the HCLs, averaged for *R* network operation rounds. From Figure 3, the red line represents UBER’s load balancing ratio performance trend under low MS altitude of 120 m, the green line represents UBER’s load balancing ratio performance trend under normal MS altitude of 230 m, and the blue line represents UBER’s load balancing ratio performance trend under high MS altitude of 340 m. With respect to Figure 3, a higher LBR value (LBR ≥ 0.5) is desired as this means data traffic coming from the HCLs is well distributed (or balanced) among the MS to avoid underloading and overloading scenarios. The network recorded an average LBR of 0.2478, 0.6466 and 0.3787 for the high-altitude (indicated as blue line), normal-altitude (indicated as green line) and low-altitude (indicated as red line) scenarios over the period of network operation. This means that by operating the MS at normal altitude (indicated as green line), the network gains LBR of 61.67% and 41.42% over a similar network configuration operating at high altitude (indicated as blue line) and low altitude (indicated as red line), respectively. The reason for this is that operating at high altitude (indicated with blue line) results in underloading (as a result of long processing delays) due to weak strength of MS coverage; while operating at low altitude (indicated with red line) results in overloading (as a result of packet flooding, frequent packet drops, reconnection and retransmissions) due to simultaneous detection of MS by multiple HCLs. Figure 3 results underscore the significance of utilizing suitable MS altitude on LBR performance.

#### 4.4.4. Effect of MS Altitude on Topology Fluctuation Effect Ratio (TFER)

**Figure 4:**
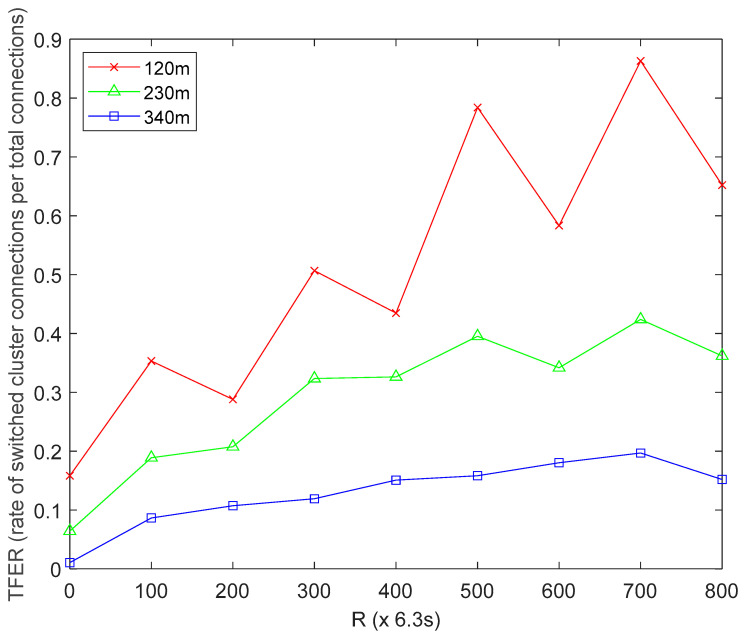
Effect of MS Altitude on Topology Fluctuation Effect Ratio.

The TFER is defined as the frequency by which the HCLs detect and switch MS connections as a ratio of the total number of connections after *R* network operation rounds. From Figure 4, the red line represents UBER’s topology fluctuation effect ratio performance trend under low MS altitude of 120 m, the green line represents UBER’s topology fluctuation effect ratio performance trend under normal MS altitude of 230 m, and the blue line represents UBER’s topology fluctuation effect ratio performance trend under high MS altitude of 340 m. With regard to Figure 4, a moderate TFER value (0.2 ≤ TFER ≤ 0.4) is desired as this measures the frequency of switching MS-to-HCL connectivity as a result of changing HC formation, location and re-assignment of HCLs. A moderate TFER value is desired to avoid under-sensitivity and oversensitivity scenarios. The network recorded an average TFER of 0.1291, 0.2925 and 0.5136 for the high-altitude (indicated as blue line), normal-altitude (indicated as green line) and low-altitude (indicated as red line) scenarios over the period of network operation. This means that by operating the MS at normal altitude (indicated as green line), the network gains TFER of 55.86% and 75.57% over a similar network configuration operating at high altitude (indicated as blue line) and low altitude (indicated as red line), respectively. The technical reason for this is that operating at high altitude (indicated with blue line) results in under-sensitivity due to near MS out-of-reach issues; while operating at low altitude (indicated with red line) results in oversensitivity to HC variations and re-configuration due to high MS proximity. Figure 4 results underline the influence of employing suitable MS altitude on TFER performance.

Table 2 summarizes UBER performance evaluation results for different MS altitudes.

### 4.5. Comparitive Performance Evaluation of UBER

To conduct a comparative performance evaluation of UBER, HYBRID and MS-DVCR are selected as baseline protocols for benchmarking in this research.

#### 4.5.1. Evaluation of Energy Consumption (ENC) Performance

**Figure 5:**
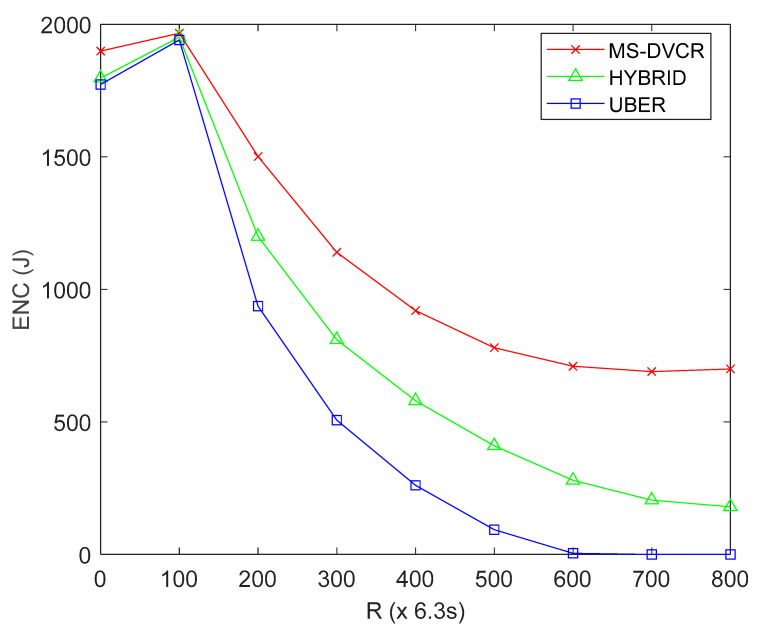
Energy Consumption Performance.

ENC is defined as the aggregate energy tax (*E*_TAX_) by the SNs after *R* network operation rounds. From Figure 5, the red line represents MS-DVCR’s energy consumption performance trend, the green line represents HYBRID’s energy consumption performance trend, and the blue line represents UBER’s energy consumption performance trend under normal MS altitude of 230 m. Figure 5 shows the comparative plot of ENC for UBER (indicated as blue line) against HYBRID (indicated as green line) and MS-DVCR (indicated as red line). UBER (indicated with blue line) recorded lesser *E*_TAX_ with 25.59% and 46.48% improvements over HYBRID (indicated with green line) and MS-DVCR (indicated with red line), respectively. The technical justification for UBER’s performance improvement is due to the energy conservation benefits from the dynamic residual energy thresholding technique, which ensures robust HCL-to-MS data transmission, and reduced energy consumption for HC setup and topology maintenance.

#### 4.5.2. Evaluation of Network Coverage (COV) Performance

**Figure 6:**
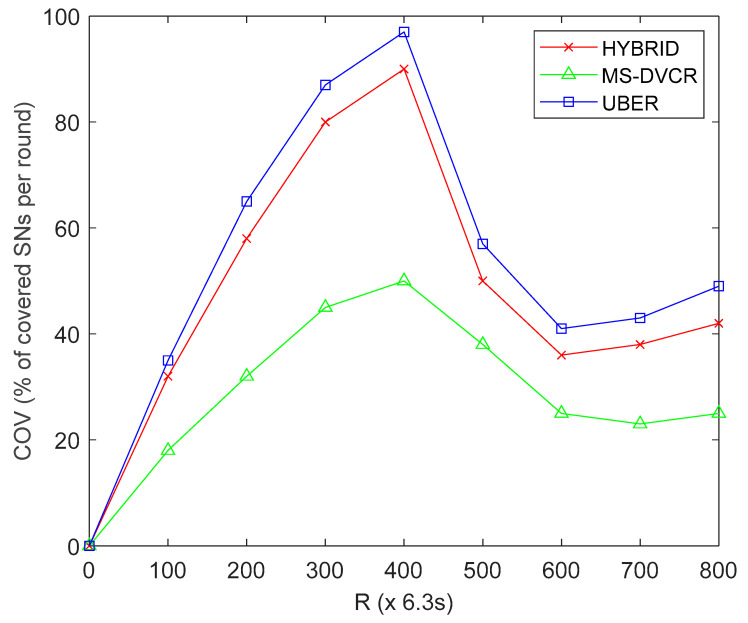
Network Coverage Performance.

COV is defined as the percentage of successfully covered SNs to the total node density for the LF network field. From Figure 6, the red line represents HYBRID’s network coverage performance trend, the green line represents MS-DVCR’s network coverage performance trend, and the blue line represents UBER’s network coverage performance trend under normal MS altitude of 230 m. Figure 6 provides the comparative plot of COV for UBER (indicated as blue line) with respect to HYBRID (indicated as red line) and MS-DVCR (indicated as green line). UBER (indicated with blue line) exhibited better network coverage by yielding improvements of 28.44% and 47.33% over HYBRID (indicated with red line) and MS-DVCR (indicated with green line), respectively. UBER’s performance enhancements are due to the effective HCL-to-MS cluster-based connectivity chain and suitable selection of MS altitude for extending network coverage during network operation.

#### 4.5.3. Evaluation of Received Packets (RPK) Performance

**Figure 7:**
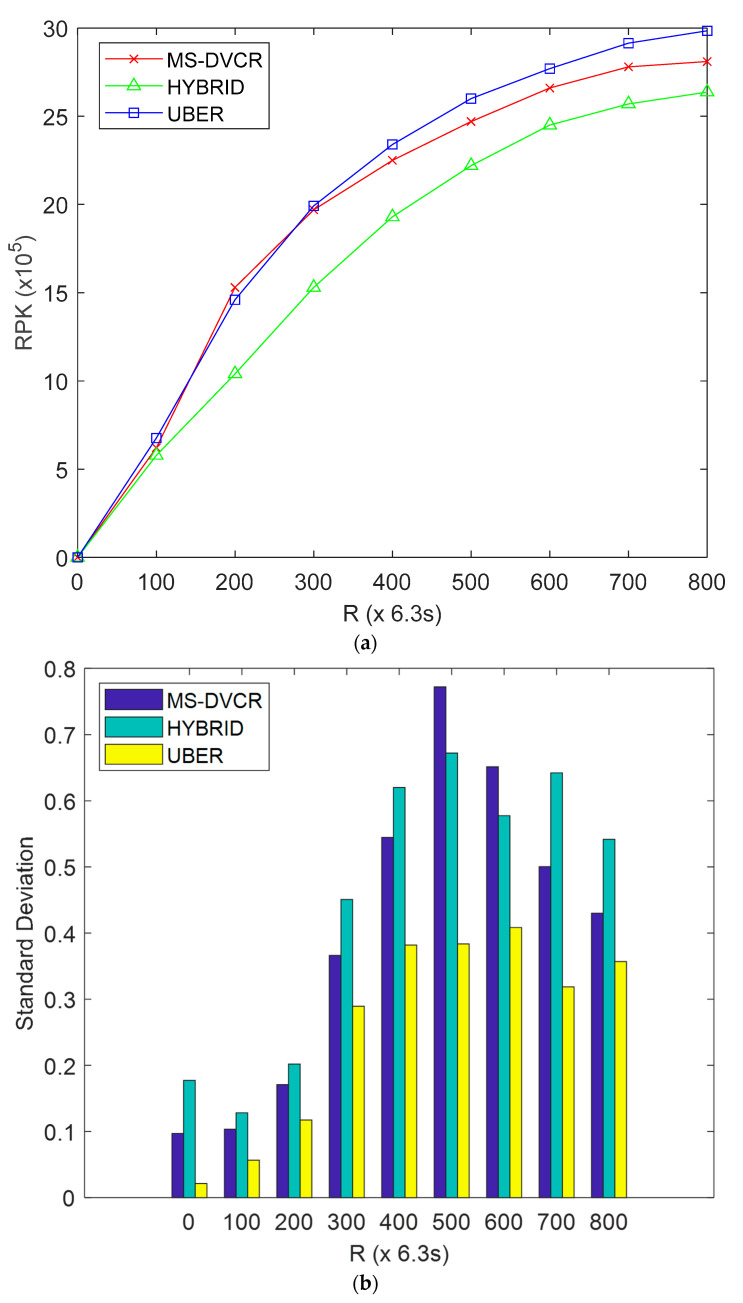
(**a**) Received Packets Performance. (**b**) Standard Deviation Performance Evaluation of SN Failures Detected (SNFD) Performance.

RPK is defined as total received packets recorded via the MS-to-LMS transmission link after *R* network operation rounds. From Figure 7a, the red line represents MS-DVCR’s received packets performance trend, the green line represents HYBRID’s received packets performance trend, and the blue line represents UBER’s received packets performance trend under normal MS altitude of 230 m. Figure 7a depicts the comparative plot of RPK for UBER (indicated as blue line) compared to HYBRID (indicated as green line) and MS-DVCR (indicated as red line). UBER (indicated with blue line) displayed higher RPK by giving improvements of 15.68% and 3.637% over HYBRID (indicated with green line) and MS-DVCR (indicated with red line), respectively. The technical justification for UBER’s performance improvements is as a result of adopting an MS-assisted data-gathering strategy, cluster resolution and assimilation for orphaned SNs, and minimal-hop SN-to-MS data transmission. The close RPK performance between UBER and MS-DVCR is simply a slight performance tradeoff and it is not as a result of high statistical error ranges or statistical dependency issues. The standard deviation for RPK performance is shown in Figure 7b to buttress this performance evaluation. The standard deviation plot demonstrates that UBER has a significantly lesser standard deviation values for RPK (≤0.4) in all instances of network operation rounds compared to MS-DVCR and HYBRID. Irrespective of the close performance between UBER and MS-DVCR, the lesser standard deviation values recorded for UBER is a relatively strong indicator of stable packet reception and statistical significance of the obtained RPK results.

**Figure 8:**
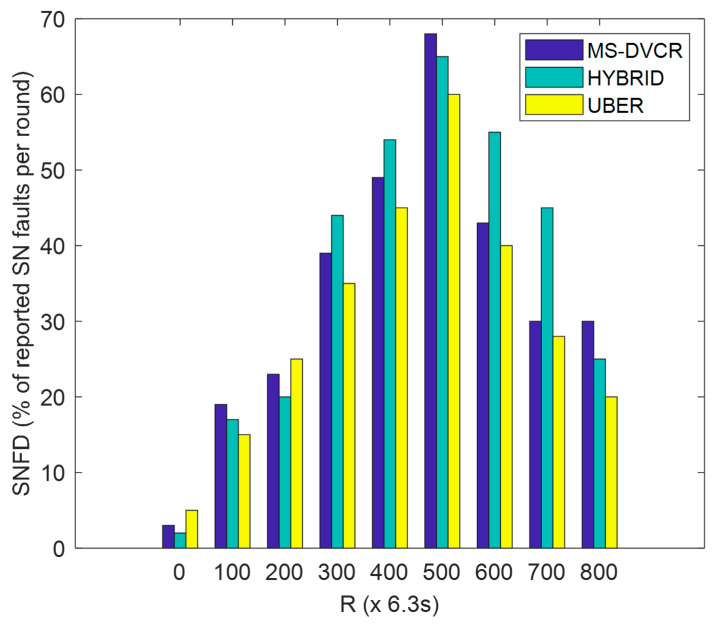
SN Failures Detected Performance.

SNFD is defined as the percentage of SN failures detected at each specified round of network operation. From Figure 8, the blue bar represents MS-DVCR’s SN failures detected performance trend, the green bar represents HYBRID’s SN failures detected performance trend, and the yellow bar represents UBER’s SN failures detected performance trend under normal MS altitude of 230 m. Figure 8 provides the comparative plot of SNFD for UBER (indicated as yellow bar) with respect to HYBRID (indicated as green bar) and MS-DVCR (indicated as blue bar). UBER (indicated with yellow bar) exhibited lower SNFD by recording gains of 19.78% and 11.35% over HYBRID (indicated with green bar) and MS-DVCR (indicated with blue bar), respectively. UBER’s performance gains are due to the effective MS-assisted network coverage and recovery mechanism and effective HCL-to-HCM enlisting process, which makes it possible to achieve seamless end-to-end data transmission with reduced SN failures.

#### 4.5.4. Evaluation of Route Failures Detected (RFD) Performance

**Figure 9:**
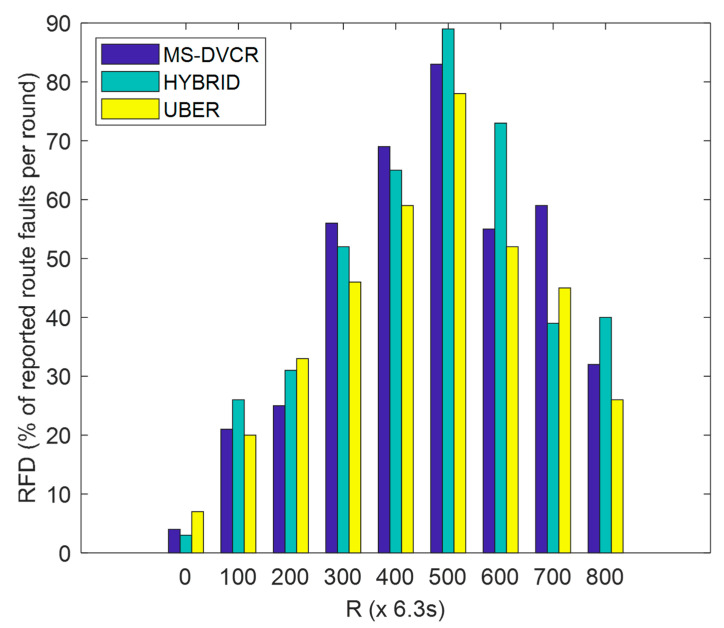
Route Failures Detected Performance.

RFD is defined as the percentage of route breakages detected at each specified round of network operation. From Figure 9, the blue bar represents MS-DVCR’s route failures detected performance trend, the green bar represents HYBRID’s route failures detected performance trend, and the yellow bar represents UBER’s route failures detected performance trend under normal MS altitude of 230 m. Figure 9 shows the comparative plot of RFD for UBER (indicated as yellow bar) against HYBRID (indicated as green bar) and MS-DVCR (indicated as blue bar). UBER (indicated with yellow bar) recorded lesser RFD by showing improvements of 46.44% and 44.89% over HYBRID (indicated with green bar) and MS-DVCR (indicated with blue bar), respectively. The technical justification for UBER’s performance improvement is due to the effective HCL-to-MS cluster-based connectivity chain and MS-based network monitoring and recovery mechanism that ensures robust HCL-to-MS data transmission with reduced route failures.

#### 4.5.5. Evaluation of Routing Overhead (ROH) Performance

**Figure 10:**
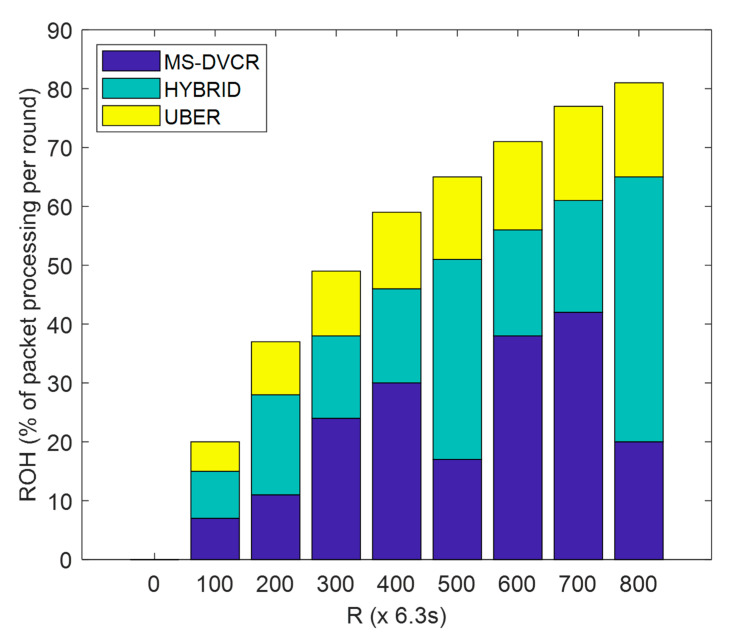
Routing Overhead Performance.

ROH is defined as the ratio (in percentage) of packet processing (prior to actual data transmission) duration to network operation duration. From Figure 10, the blue bar segment represents MS-DVCR’s routing overhead performance trend, the green bar segment represents HYBRID’s routing overhead performance trend, and the yellow bar segment represents UBER’s routing overhead performance trend under normal MS altitude of 230 m. Figure 10 provides the comparative plot of ROH for UBER (indicated as yellow bar segment) with respect to HYBRID (indicated as green bar segment) and MS-DVCR (indicated as blue bar segment). UBER (indicated with yellow bar segment) exhibited lower ROH by recording gains of 29.38% and 16.45% over HYBRID (indicated with green bar segment) and MS-DVCR (indicated with blue bar segment), respectively. UBER’s performance gains are due to the reduced algorithmic complexity, which makes it possible for the network to construct routes and carry out routing operations with reduced computational costs.

#### 4.5.6. Evaluation of End-To-End Delay (ETE) Performance

**Figure 11:**
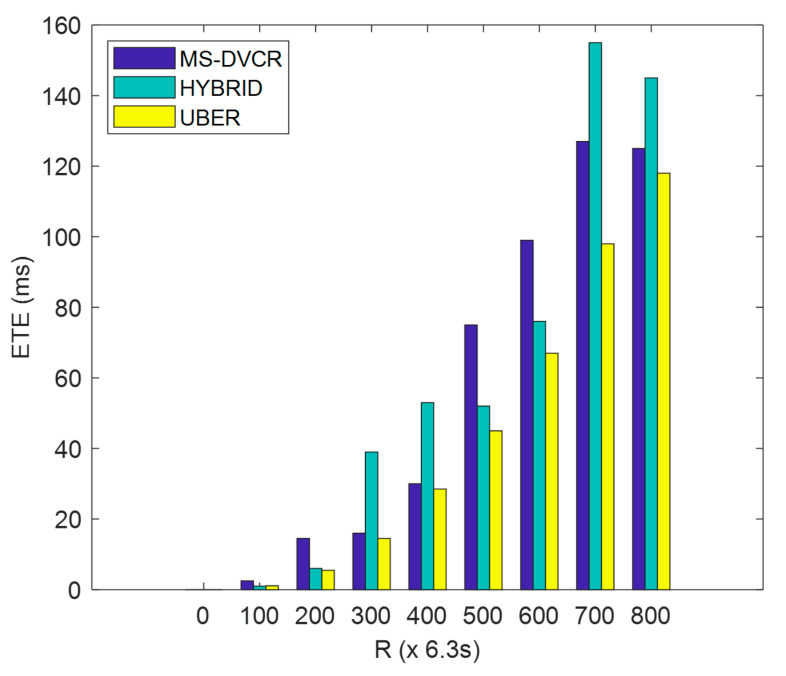
End-to-End Delay Performance.

ETE is defined as the total duration measured from initial packet generation at the HCM to eventual delivery via the MS-to-LMS transmission link. From Figure 11, the blue bar represents MS-DVCR’s end-to-end delay performance trend, the green bar represents HYBRID’s end-to-end delay performance trend, and the yellow bar represents UBER’s end-to-end delay performance trend under normal MS altitude of 230 m. Figure 11 depicts the comparative plot of ETE for UBER (indicated as yellow bar) in comparison to HYBRID (indicated as green bar) and MS-DVCR (indicated as blue bar). UBER (indicated with yellow bar) displayed lesser ETE by 58.56% and 54.33% improvements over HYBRID (indicated with green bar) and MS-DVCR (indicated with blue bar), respectively. The technical justification for UBER’s performance improvements is due to the adaptive MS-assisted maintenance of HCM-to-HCL, HCL-to-MS, and MS-to-LMS data forwarding chains. This adaptive nature makes it possible to preserve end-to-end data transmission links with lesser delays.

Table 3 summarizes UBER’s comparative performance evaluation against HYBRID and MS-DVCR.

## 5. Conclusions

This paper treats the issues of loss of coverage and rapid energy drainage for wireless livestock sensor network applications by developing a UAV-based energy-efficient reconfigurable routing (UBER) scheme for smart wireless livestock sensor networking. This research contrived and incorporated a dynamic residual energy thresholding mathematical model, robust cluster-to-UAV link formation strategy, and UAV-assisted network coverage and recovery mechanisms into this scheme for the proposed integrated heterogenous network model. Experiments were carried out with OMNET++ and MATLAB. The performance of UBER was analyzed using low, normal, and high UAV altitude scenarios. Simulation results revealed that operating the network with a UAV altitude at normal range yielded performance gains with respect to network stability, load balancing ratio, and topology fluctuation effect ratio. A comparative performance analysis of UBER was performed with respect to HYBRID and MS-DVCR. With regard to the obtained results, UBER recorded significant performance improvements in terms of energy consumption, network coverage, received packets, SN failures detected, route failures detected, routing overhead, and end-to-end delay. The results obtained demonstrated that the UBER scheme is highly efficient with competitive performance against the benchmarked CBR schemes. Future research work will focus on developing a lightweight, reliable, and energy-efficient repair/recovery scheme for the identified SNFD and RFD issues.

## Figures and Tables

**Table 1:** Simulation Parameters.

Symbol	Description	Value
SN-DEP	SNs Deployed	250
LF-NS	LF Network Size	2000 m × 2000 m
PKS	Packet Size	500 bytes
*E*_TAX_ TL	Energy Tax Threshold Levels	8
*E*_tot_	Total Energy of each SN (before depletion)	2 J
*E*_idle_	Idle Energy	0.2 μJ
*E*_agg_	Aggregation Energy	5 pJ/bit
*E*_EC_	Electronic Circuitry Energy	5 nJ/bit
*CTR*_max_	Maximum Transmission Range	250 m
*A*	Path Loss Exponent	2.5
*SN-RS*	SN Receiver Sensitivity	−95 dBm
*MS-ALT*	MS Maximum Altitude	230 m
*MS-V*	MS Velocity	20 m/s
*MS-SR*	MS Signaling Rate	2 s
*MS-TD*	MS Tour Duration	960 s
*AVG-STAT*	Simulation Runs for Statistical Averaging	50

**Table 2:** Summary of UBER Performance with MS Altitude Variations.

	% Gain of 230 M Over	
Metric	340 M	120 M
NST	31.58%	12.79%
LBR	61.67%	41.42%
TFER	55.86%	75.57%

**Table 3:** Summary of UBER Comparative Performance Results.

Metric	HYBRID	MS-DVCR
ENC	25.59%	46.48%
COV	28.44%	47.33%
RPK	15.68%	3.637%
SNFD	19.78%	11.35%
RFD	46.44%	44.89%
ROH	29.38%	16.45%
ETE	58.56%	54.33%

## References

[1] Alanezi M.A., Shahriar M.S., Hasan M.B., Ahmed S., Sha’aban Y.A., Bouchekara H.R.E.H. (2022). Livestock Management with Unmanned Aerial Vehicles: A Review. IEEE Access.

[2] Barbedo J.G.A., Koenigkan L.V. (2018). Perspectives on the use of unmanned aerial systems to monitor cattle. Outlook Agric..

[3] Awan K.M., Sherazi H.H.R., Ali A., Iqbal R., Khan Z.A., Mukherjee M. (2019). Energy-aware cluster-based routing optimization for WSNs in the livestock industry. Trans. Emerg. Telecommun. Technol..

[4] Maddikunta P.R., Hakak S., Alazab M., Bhattacharya S., Gadekallu T.R., Khan W.Z., Pham Q.V. (2021). Unmanned Aerial Vehicles in Smart Agriculture: Applications, Requirements, and Challenges. IEEE Sens. J..

[5] Friha O., Ferrag M.A., Shu L., Maglaras L., Wang X. (2021). Internet of Things for the Future of Smart Agriculture: A Comprehensive Survey of Emerging Technologies. IEEE/CAA J. Autom. Sin..

[6] Long N.K., Sammut K., Sgarioto D., Garratt M., Abbass H.A. (2020). A Comprehensive Review of Shepherding as a Bio-Inspired Swarm-Robotics Guidance Approach. IEEE Trans. Emerg. Top. Comput. Intell..

[7] Xiang T.-Z., Xia G.-S., Zhang L. (2019). Mini-Unmanned Aerial Vehicle-Based Remote Sensing: Techniques, applications, and prospects. IEEE Geosci. Remote Sens. Mag..

[8] Boursianis A.D., Papadopoulou M.S., Diamantoulakis P., Liopa-Tsakalidi A., Barouchas P., Salahas G., Karagiannidis G., Wan S., Goudos S. (2020). KInternet of Things (IoT) and Agricultural Unmanned Aerial Vehicles (UAVs) in smart farming: A comprehensive review. Internet Things.

[9] Kakamoukas G., Sariciannidis P., Livanos G., Zervakis M., Ramnalis D., Polychronos V., Karamitsou T., Folinas A., Tsitsiokas N. A Multi-collective, IoT-enabled, Adaptive Smart Farming Architecture. Proceedings of the 2019 IEEE International Conference on Imaging Systems and Techniques (IST).

[10] Urdaneta G.A., Meyers C., Rogalski L. (2022). How do drones facilitate human life?. Future Technol..

[11] Mistry C., Ghosh A., Biswas M., Basak B.B.A. (2022). Applications of Internet of Things and Unmanned Aerial Vehicle in Smart Agriculture: A Review. OSF Prepr..

[12] Casas R., Hermosa A., Marco Á., Blanco T., Zarazaga-Soria F.J. (2021). Real-Time Extensive Livestock Monitoring Using LPWAN Smart Wearable and Infrastructure. Appl. Sci..

[13] Tahir A., Böling J., Haghbayan M.-H., Toivonen H.T., Plosila J. (2019). Swarms of Unmanned Aerial Vehicles—A Survey. J. Ind. Inf. Integr..

[14] Pajares G. (2015). Overview and Current Status of Remote Sensing Applications Based on Unmanned Aerial Vehicles (UAVs). Photogramm. Eng. Remote Sens..

[15] Mukhamediev R.I., Symagulov A., Kuchin Y., Zaitseva E., Bekbotayeva A., Yakunin K., Assanov I., Levashenko V., Popova Y., Akzhalova A. (2021). Review of Some Applications of Unmanned Aerial Vehicles Technology in the Resource-Rich Country. Appl. Sci..

[16] Elmokadem T., Savkin A.V. (2021). Towards Fully Autonomous UAVs: A Survey. Sensors.

[17] Sivakumar M., TYJ N.M. (2021). A Literature Survey of Unmanned Aerial Vehicle Usage for Civil Applications. J. Aerosp. Technol. Manag..

[18] Petrova T., Petrov Z. (2021). Analysis of Efficiency of the Unmanned Aerial Vehicles Use in Contemporary Agrotechnologies. Int. J. Inf. Technol. Secur..

[19] Freed T., Carson V.C., Doerr K.H. (2021). Optimizing a RFID-UAV cattle search tour. Int. J. RF Technol..

[20] Chamoso P., Raveane W., Parra V., González A. (2014). UAVs Applied to the Counting and Monitoring of Animals. Ambient Intelligence—Software and Applications.

[21] Afrianto I., Wahjuni S., Djatna T. (2020). Model of Ubiquitous Precision Livestock System 4.0: A Technological Review. FoITIC.

[22] Chabot D., Bird D.M. (2015). Wildlife research and management methods in the 21st century: Where do unmanned aircraft fit in?. J. Unmanned Veh. Syst..

[23] Rivas A., Chamoso P., González-Briones A., Corchado J.M. (2018). Detection of Cattle Using Drones and Convolutional Neural Networks. Sensors.

[24] Behjati M., Noh A.B.M., Alobaidy H.A.H., Zulkifley M.A., Nordin R., Abdullah N.F. (2021). LoRa Communications as an Enabler for Internet of Drones towards Large-Scale Livestock Monitoring in Rural Farms. Sensors.

[25] Xu J., Solmaz G., Rahmatizadeh R., Turgut D., Bölöni L. Animal monitoring with unmanned aerial vehicle-aided wireless sensor networks. Proceedings of the 2015 IEEE 40th Conference on Local Computer Networks (LCN).

[26] Xu J., Solmaz G., Rahmatizadeh R., Turgut D., Boloni L. (2016). Internet of Things Applications: Animal Monitoring with Unmanned Aerial Vehicle. arXiv.

[27] Heinzelman W.R., Chandrakasan A., Balakrishnan H. Energy-efficient communication protocol for wireless microsensor networks. Proceedings of the 33rd Annual Hawaii International Conference on System Sciences.

[28] Heinzelman W.B., Chandrakasan A.P., Balakrishnan H. (2002). An application-specific protocol architecture for wireless microsensor networks. IEEE Trans. Wirel. Commun..

[29] Bandyopadhyay S., Coyle E.J. An energy efficient hierarchical clustering algorithm for wireless sensor networks. Proceedings of the IEEE INFOCOM 2003—Twenty-Second Annual Joint Conference of the IEEE Computer and Communications Societies.

[30] Huang H., Wu J. A probabilistic clustering algorithm in wireless sensor networks. Proceedings of the VTC-2005-Fall—2005 IEEE 62nd Vehicular Technology Conference.

[31] Sivakumar R., Sinha P., Bharghavan V. (1999). CEDAR: A core-extraction distributed ad hoc routing algorithm. IEEE J. Sel. Areas Commun..

[32] Ding P., Holliday J., Celik A. (2005). Distributed Energy-Efficient Hierarchical Clustering for Wireless Sensor Networks. Distributed Computing in Sensor Systems.

[33] Neethirajan S. (2017). Recent advances in wearable sensors for animal health management. Sens. Bio-Sens. Res..

[34] Lotfinezhad M., Liang B. Energy efficient clustering in sensor networks with mobile agents. Proceedings of the IEEE Wireless Communications and Networking Conference.

[35] Morsly Y., Aouf N., Djouadi M.S. Dynamic decentralized/centralized free conflict UAV’s team allocation. Proceedings of the 2012 IEEE International Instrumentation and Measurement Technology Conference Proceedings.

[36] Behera T.M., Mohapatra S.K., Samal U.C., Khan M.S. (2019). Hybrid heterogeneous routing scheme for improved network performance in WSNs for animal tracking. Internet Things.

[37] Rahmatizadeh R., Khan S.A., Jayasumana A.P., Turgut D., Bölöni L. Routing towards a mobile sink using virtual coordinates in a wireless sensor network. Proceedings of the 2014 IEEE International Conference on Communications (ICC).

[38] Rahman G.M.E., Wahid K.A. (2022). LDCA: Lightweight Dynamic Clustering Algorithm for IoT-Connected Wide-Area WSN and Mobile Data Sink Using LoRa. IEEE Internet Things J..

[39] Wark T., Crossman C., Hu W., Guo Y., Valencia P., Sikka P., Corke P., Lee C., Henshall J., Prayaga K. The Design and Evaluation of a Mobile Sensor/Actuator Network for Autonomous Animal Control. Proceedings of the 2007 6th International Symposium on Information Processing in Sensor Networks.

[40] Gu J., Su T., Wang Q., Du X., Guizani M. (2018). Multiple Moving Targets Surveillance Based on a Cooperative Network for Multi-UAV. IEEE Commun. Mag..

[41] Hu J., Turgut A.E., Krajnik T., Lennox B., Arvin F. (2022). Occlusion-Based Coordination Protocol Design for Autonomous Robotic Shepherding Tasks. IEEE Trans. Cogn. Dev. Syst..

[42] Lin C., Han G., Qi X., Du J., Xu T., Martínez-García M. (2021). Energy-Optimal Data Collection for Unmanned Aerial Vehicle-Aided Industrial Wireless Sensor Network-Based Agricultural Monitoring System: A Clustering Compressed Sampling Approach. IEEE Trans. Ind. Inform..

[43] Gnanasekera M., Katupitiya J., Savkin A.V., de Silva A.H.T.E. (2021). A Range-Based Algorithm for Autonomous Navigation of an Aerial Drone to Approach and Follow a Herd of Cattle. Sensors.

[44] Salami A.F., Anwar F., Priantoro A.U. (2009). An investigation into clustering routing protocols for wireless sensor networks. Sens. Transducers.

[45] Salami A.F., Bari S.M.S., Anwar F., Khan S. Feasibility analysis of clustering routing protocols for multipurpose sensor networking. Proceedings of the 2nd International Conference on Multimedia and Computational Intelligence (ICMCI).

[46] Astakhova T. Research on the Energy Characteristics of Routing in Wireless Sensor Networks. In CEUR Workshop Proceedings 2020. http://ceur-ws.org/Vol-2590/short15.pdf.

[47] Li X., Huang H., Savkin A.V., Zhang J. (2022). Robotic Herding of Farm Animals Using a Network of Barking Aerial Drones. Drones.

[48] Yaxley K.J., Joiner K.F., Abbass H. (2021). Drone approach parameters leading to lower stress sheep flocking and movement: Sky shepherding. Sci. Rep..

[49] Salami A.F., Anwar F., Aibinu A.M., Bello-Salau H., Abdalla A.H. Investigative analysis of clustering routing protocols for scalable sensor networks. Proceedings of the 4th IEEE International Conference on Mechatronics (ICOM).

[50] Bello-Salau H., Salami A.F., Anwar F., Aibinu A.M. Evaluation of Radio Propagation Techniques for Hierarchical Sensor Networks. Proceedings of the 4th IEEE International Conference on Mechatronics (ICOM).

[51] Bello-Salau H., Salami A.F., Anwar F., Islam M.R. (2011). Analysis of radio model performance for clustering sensor networks. Sens. Transducers.

[52] Friis H.T. (1946). A Note on a Simple Transmission Formula. Proc. IRE.

[53] (2020). IEEE Standard for Low-Rate Wireless Networks.

[54] 4 GHz IEEE 802.15.4/ZigBee-ready RF Transceiver Applications. https://www.ti.com/product/CC2420.

